# Effect of cotton padding on the setting properties of plaster slabs

**DOI:** 10.1308/003588413X13511609956291

**Published:** 2013-03

**Authors:** D Barlow, R Bansal, T Barlow, SJ Rhee, JH Kuiper, NK Makwana

**Affiliations:** ^1^Robert Jones and Agnes Hunt Orthopaedic Hospital NHS Foundation Trust, UK; ^2^University Hospitals Coventry and Warwickshire NHS Trust, UK

**Keywords:** Plaster of Paris, Strength, Modification of application

## Abstract

**Introduction:**

Plaster of Paris (PoP) impregnated bandages have been used to maintain the position of bones and joints for over a century. Classically, wool dressing is applied to the limb before the PoP, which can then be moulded to the desired shape. A modification of this practice is to wrap the PoP bandages circumferentially in cotton before wetting and applying to the patient in an attempt to reduce inhalation of plaster dust and reduce mess. However, this may affect the water content of the cast and therefore also its setting properties and strength. This study compared the setting properties of PoP casts when used with and without cotton wrapping.

**Methods:**

Sixty specimens, compliant with the American Society for Testing and Materials standards for three-point bending tests, were prepared, with thirty wrapped in cotton. All were weighed before and after water immersion, and wrapped around a plastic cylinder to mimic limb application. Bending stiffness and yield strength was measured on a servohydraulic materials testing machine at 2, 6, 12, 24, 48 and 72 hours.

**Results:**

The water content of cotton-wrapped plaster was significantly higher (50%) than that of standard plaster. It had significantly lower strength up to 24 hours and significantly lower stiffness up to 72 hours.

**Conclusions:**

The initial decrease in strength and stiffness of the cast wrapped in cotton may comprise the ability of the backslab to hold the joint or bone in an optimal position. Any modification of the standard plaster slab application technique should allow for the potential adverse effects on the plaster setting properties.

Plaster of Paris (PoP) has been used in the treatment of fractures since the 1800s. Antonius Mathijsen used linen, into which he rubbed PoP, which was then hardened using water to splint broken limbs.^1^

A plaster cast is made from a cotton-type dressing that is impregnated with PoP, so named after the large gypsum deposits in Paris from which the plaster is made. PoP or calcined gypsum is a fine powder. When water is added, the more soluble form of calcium sulphate returns to the relatively insoluble crystalline form in an exothermic reaction:^2^

2 (CaSO_4_·½ H_2_O) + 3 H_2_O ® 2 (CaSO_4_.2H_2_O) + Heat

The PoP in the bandages usually sets completely in 45 minutes but is not completely dry for 71 hours^3^ and is not recommended for weight bearing for 72 hours.^4^

PoP bandages are still used routinely in emergency departments and orthopaedic units to hold joints and bones in position, especially after manipulation or surgery. PoP has advantages over resin impregnated fibreglass in terms of malleability, conformability and cost.^4^ Commonly, it is used in the form of a backslab as it can be moulded easily to produce a comfortable fit. Classically, a wool dressing is applied around the skin. PoP bandages are dipped in water, squeezed to remove the excess water, then applied in layers around the wool dressing. This can then be moulded to the appropriate position, forming a cast. A bandage is applied to complete the backslab, which can be done before or after moulding.

A modification of this practice is to wrap the PoP bandages circumferentially in cotton or orthopaedic wool before wetting, squeezing and applying to the patient. The wrapping covers and holds the plaster dust while handling, dipping and squeezing the slab, reducing the risk of inhalation of plaster dust. This method also creates less plaster splash once the cast is wet. However, the wrapping will hold moisture and this modification of standard practice may therefore affect the water content of the plaster cast and, consequently, its setting properties and resulting strength. In previous studies, water immersion has been shown to significantly decrease the strength of PoP bandages.^4–6^ The aim of this biomechanical study was to compare stiffness and strength of PoP when used with and without cotton wrapping.

## Methods

Specimens were prepared in the form of 150mm × 22mm sized slabs that had a thickness of ten layers of PoP bandage. The thickness of each specimen was measured at three points to obtain an average value. The dimensions were chosen to comply with the 2007 standard for three-point bending tests by the American Society for Testing and Materials (ASTM).^7^

Six sets of specimens were prepared, each comprising five slabs with and five slabs without a cotton layer. They were dipped for 4–5 seconds in water at 24°C. The excess water was squeezed out before the specimens were bandaged onto cotton-wrapped plastic cylinders to mimic application to a limb. Each slab was weighed before and after being soaked in water.

Bending stiffness and bending yield strength of the plaster slabs was measured at 2, 6, 12, 24, 48 and 72 hours at room temperature using the standard ASTM 2007 3-point bending test performed on a servohydraulic materials testing machine (ESH Testing, Brierley Hill, UK). Force and displacement during the test were digitised and stored on a computer for further analysis. Tangential bending stiffness and bending strength were determined from the force displacement data recorded on the computer, in accordance with the ASTM 2007 standards.^7^ Statistical analysis was performed using a two-sided t-test with statistical significance defined as *p*<0.05.

## Results

**Figure 1:**
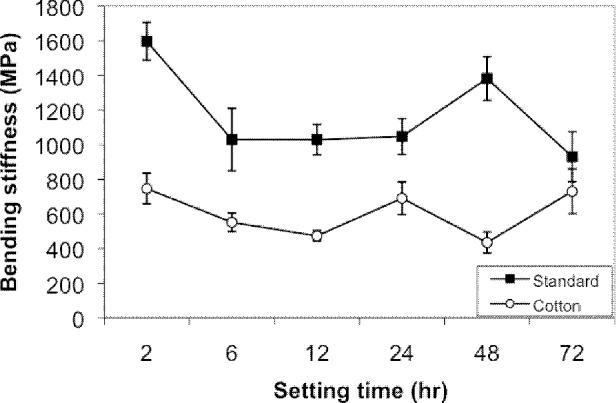
Mean stiffness of plaster cast against time

**Figure 2:**
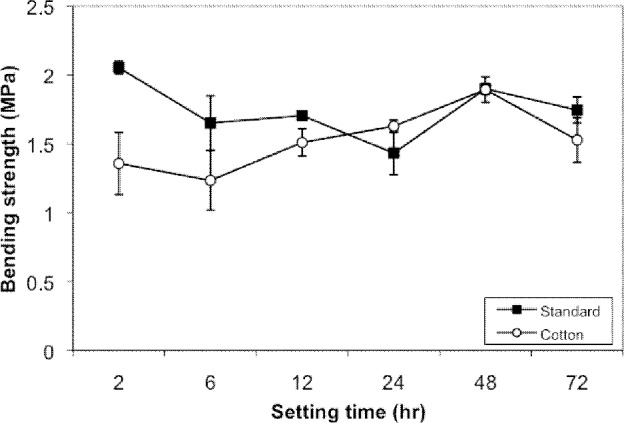
Mean strength of plaster cast against time

The water content of the plaster with a cotton layer was 50% larger than that of plaster without cotton (*p*<0.05) (Table 1). Strength of standard setting slabs (without cotton) was consistent and had a minimum value of 1.5MPa for all time periods. Strength of the moist setting slabs (with cotton) was less consistent at early time periods and ranged between 1MPa and 2MPa. No difference in strength was found after 24 hours. Stiffness of standard setting slabs was higher up to 24 hours but after 48 hours no difference was found. Table 2 summarises the setting properties at each of the time settings with standard errors. The mean stiffness and strength of the slabs against time are shown in Figures 1 and 2.

**Table 1:** Proportion of water to plaster by weight

	Percentage of water to plaster
Without cotton layer (*n*=30)	98% (range: 86–105%)
With cotton layer (*n*=30)	146% (range: 140–156%)

**Table 2:** Setting properties at each time setting

Time	Bending stiffness (MPa)				Bending strength (MPa)			
	Without cotton	Standard error	With cotton	Standard error	Without cotton	Standard error	With cotton	Standard error
2 hrs	1,597.58	108.91	746.95*	88.26	2.05	0.05	1.36*	0.23
6 hrs	1,029.96	180.65	552.36*	53.24	1.65	0.20	1.23*	0.22
12 hrs	1,029.54	87.64	473.73*	30.52	1.71	0.03	1.51*	0.10
24 hrs	1,048.02	103.77	691.22*	94.14	1.43	0.16	1.63	0.04
48 hrs	1,382.77	126.24	435.70*	61.33	1.90	0.04	1.89	0.09
72 hrs	931.07	144.34	730.96	129.19	1.75	0.09	1.53	0.16

* *p*<0.05

## Discussion

The ideal properties of a backslab are high strength, low weight, rapid setting time, malleability, high radiolucency and affordability.^8^ Such features enable the cast to achieve its function of protection, support and holding the limb or joint in a position of function. The required mechanical properties of PoP demand high strength and elastic modulus in order to achieve these functions.

The strength of the cast derives directly from crystallisation of the gypsum and is determined by several factors:

> the quality of the material
> the water-to-gypsum ratio
> product age
> storage conditions

With a higher water-to-gypsum ratio, the strength of gypsum decreases (ie the strength of gypsum decreases with increasing water content). High strength is therefore achieved by adding to the gypsum only the amount of water needed for setting and crystallisation.^8^

Mechanical properties of gypsum and fibreglass properties have been explored previously. Wytch *et al* compared traditional PoP bandages with several polyurethane impregnated bandages.^4^ They showed that PoP was rigid but not very strong. Additionally, when immersed in water, the synthetic materials showed decreased stiffness to 53% but could recover, unlike the PoP bandages, which could not resist mechanical loading after water immersion. PoP, however, is still preferred for manipulation of fractures and joints as it is more malleable.

Berman and Parks showed that PoP materials failed in a brittle and catastrophic manner leading to severe cracking, and therefore a failure to maintain their function.^5^ They also noted that mechanical strengths and resistance were degraded by water in both synthetics and PoP, with the synthetics again being more resistant to strength degradation. Wytch *et al* echoed similar findings in their later paper and gypsum casts are not recommended for weight bearing until 72 hours after application.^6^

Our study demonstrated that the proportion of water content in the plaster bandage is altered if modifications such as wrapping a layer of cotton padding around the plaster slab are used. Other causes of increased water content occur if the plaster is not squeeze dried adequately or if thick layers of wet bandages are employed.

The cotton-wrapped plaster has significantly lower strength than the standard cast until 24 hours. After this time, there is no statistical difference between the two types of cast. This is probably related to the higher water-to-gypsum ratio, leading to slower setting and resulting in a softer cast. In our study, we found that ****the cotton padding wrapped around the plaster increased the proportion of water to plaster by nearly 50%.

The increase in the proportion of water in the cotton-wrapped cast was accompanied by less stiffness. For the standard cast, there is a dip in stiffness after 2 hours and then it tends to level out to approximately 1,000MPa. For cotton-wrapped casts, the stiffness again drops after two hours but to a level significantly lower than that of standard casts. It is not until 72 hours have passed that the stiffness of the two types of cast is similar.

## Conclusions

The cast wrapped in cotton has decreased strength and stiffness in the first 1–3 days following application. It is therefore more likely to compromise the primary role of the backslab, namely to hold the joint or bone in an optimal position. If it is stressed, it is more likely to crack and undergo brittle failure. This could have clinical consequences such as Colles manipulation losing position or hand splints losing the Edinburgh position. Any deviation from the standard plaster slab application technique should take into account the potential adverse effects of the change in the proportion of water on the plaster setting properties.
